# The mevalonate pathway in *C. Elegans*

**DOI:** 10.1186/1476-511X-10-243

**Published:** 2011-12-28

**Authors:** Manish Rauthan, Marc Pilon

**Affiliations:** 1 Department of Cell and Molecular Biology, University of Gothenburg, S-405 30 Gothenburg, Sweden

**Keywords:** *C. elegans*, mevalonate pathway, statin, coenzyme Q, dolichol, cholesterol, protein prenylation

## Abstract

The mevalonate pathway in human is responsible for the synthesis of cholesterol and other important biomolecules such as coenzyme Q, dolichols and isoprenoids. These molecules are required in the cell for functions ranging from signaling to membrane integrity, protein prenylation and glycosylation, and energy homeostasis. The pathway consists of a main trunk followed by sub-branches that synthesize the different biomolecules. The majority of our knowledge about the mevalonate pathway is currently focused on the cholesterol synthesis branch, which is the target of the cholesterol-lowering statins; less is known about the function and regulation of the non-cholesterol-related branches. To study them, we need a biological system where it is possible to specifically modulate these metabolic branches individually or in groups. The nematode *Caenorhabditis elegans *(*C. elegans*) is a promising model to study these non-cholesterol branches since its mevalonate pathway seems very well conserved with that in human except that it has no cholesterol synthesis branch. The simple genetic makeup and tractability of *C. elegans *makes it relatively easy to identify and manipulate key genetic components of the mevalonate pathway, and to evaluate the consequences of tampering with their activity. This general experimental approach should lead to new insights into the physiological roles of the non-cholesterol part of the mevalonate pathway. This review will focus on the current knowledge related to the mevalonate pathway in *C. elegans *and its possible applications as a model organism to study the non-cholesterol functions of this pathway.

## 1. Introduction

The main trunk of the mevalonate pathway is conserved throughout the animal kingdom. The mevalonate pathway converts acetyl-CoA to farnesyl diphosphate, and produces precursors for several metabolically important molecules as well as physiologically important end-products, including: 1) isopentenyl adenosine (important for t-RNA modification); 2) coenzyme Q (an antioxidant also important in the electron transport chain in mitochondria); 3) farnesyl diphosphate and geranylgeranyl diphosphate (lipid moieties that can be added to proteins to promote membrane association); 4) dolichol and dolichol-phosphate (important for protein glycosylation); and 5) cholesterol (precursor for bile acids and steroid hormones) [1]. Many enzymes catalyze the multistep pathway and its various branches, and several therapeutic inhibitors exist that target some of these enzymes, including statins (inhibitors of HMG-CoA reductase typically prescribed to lower blood cholesterol) and bisphosphonates (inhibitors of farnesyl diphosphate synthase typically prescribed to prevent bone resorption). These therapeutics have several unexplained or undesired effects, and are also likely to find novel medical indications as their effects become better understood. For example, it has been suggested that statins could have anticancer properties [2], could be used to combat multidrug resistance during chemotherapeutic cancer treatments [3], be used to improve the success of hematopoietic transplantations [4], and even delay the progress of neurological disorders such as Alzheimer disease [5].

This review will summarize what is known about the mevalonate pathway in *C. elegans*, with the aim that this model organism may emerge as a useful tool to further our understanding of this pathway, and discover novel applications for its pharmacological modulation. An interesting feature of using *C. elegans *for the study of the mevalonate pathways is that it lacks the cholesterol-synthetic branch present in mammals, instead relying on dietary sources of sterols. *C. elegans *is therefore well-suited to study the non-cholesterol branches of the pathway, which are well conserved. Our previous research has already shown that inhibiting the mevalonate pathway using statins in *C. elegans *results in embryonic lethality, larval arrest and adult sterility, and also causes mislocalization of proteins that require isoprenylation for their membrane targeting [6]. These findings suggest that the mevalonate pathway is necessary for worm survival, and that its non-cholesterol functions are evolutionary conserved.

## 2. The mevalonate pathway in *C. elegans*

### 2.1. Main trunk

**Figure 1:**
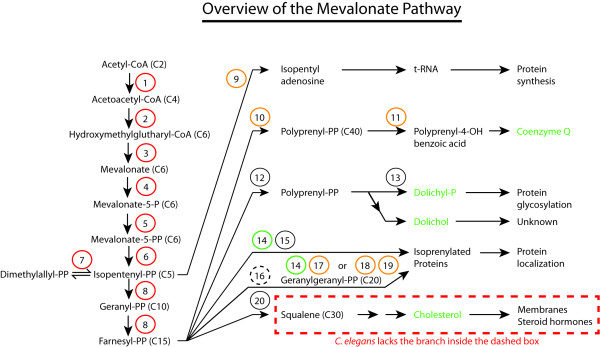
**Overview of the mevalonate pathway**. Circles represent the enzymes listed in Table 1. Red: RNAi causes embryonic lethality; Orange: RNAi causes severe phenotypes; Green: RNAi causes mild phenotypes; and Black: RNAi causes no phenotype. Biosynthetic products written in green are also exogenously supplied by the *E. coli *diet.

The main trunk of the mevalonate pathway (see Figure 1) consists of the steps through which acetyl-CoA is gradually transformed into the 5-carbon molecule isopentenyl-PP (IPP), then on to the 15-carbon molecule farnesyl-PP (FPP). The enzymes involved in these steps have been extensively reviewed; see for example Miziorko's recent survey [7].

It is clear from Table 1 that the mevalonate pathway in *C. elegans *is essential in the laboratory: RNAi knock-down of any one of the eight enzymes that belong to the main trunk of the pathway cause embryonic lethality (1-8 in Table 1). This is valuable information since it indicates that the laboratory diet of *E. coli *strain OP50 does not provide sufficient amounts of any of the metabolites ranging from acetoacetyl-CoA to FPP. Consistently, inhibiting the mevalonate pathway at the rate limiting step of HMG-CoA reductase (HMGR) using statins also causes embryonic lethality, which can be rescued by providing mevalonate exogenously (indeed essentially all effects of statins in *C. elegans *are prevented by mevalonate, demonstrating the absence of off-target effects) [6].

**Table 1:** Enzymes of the mevalonate pathway and their mutant or RNAi phenotypes in *C. elegans*.

No	Human Enzyme Name (ID)	BLAST p value	*C. elegans *ORF	*C. elegans gene*	Phenotype	**Ref**.
1	Acetyl-CoA transferase 1 (ACAT1)	1e-115	T02G5.8	*kat-1*	Embryonic lethal (RNAi); Excess fat and premature aging (mutants)	[31,33,45,46]
2	HMG-CoA synthase (HMGCS1)	2e-88	F25B4.6		Embryonic lethal (RNAi)	[31,33,47]
3	HMG-CoA reductase (HMGCR)	1e-111	F08F8.2		Embryonic lethal (RNAi); embryonic lethality, unfolded protein response activation, mislocalized prenylation reporter (statin treatment)	[7,31-33]
4	Mevalonate kinase (MVK)	3e-51	Y42G9A.4	*mvk-1*	Embryonic lethal (RNAi)	[31,32]
5	Phosphomevalonate kinase (PMVK)	5e-33	F32D8.13		Embryonic lethal (RNAi)	[32,33]
6	Mevalonate (diphospho) decarboxylase (MVD)	2e-61	Y48B6A.13		Embryonic lethal (RNAi)	[48]
7	Isopentenyl diphosphate isomerase (IDI1)	7e-32	K06H7.9	*idi-1*	Embryonic lethal (RNAi); larval paralysis and lethality, defect in engulfment of apoptotic corpses (mutant)	[8,33]
8	Farnesyl diphosphate synthase (FDPS)	4e-52	R06C1.2	*fdps-1*	Embryonic lethal (RNAi)	[33]
9	tRNA isopentenyldiphosphate transferase (TRIT1)	9e-49	ZC395.6	*gro-1*	Maternal sterility (RNAi); deregulated developmental, behavioral, and reproductive rates, as well as increased life span (mutant)	[11,33]
10	prenyl (decaprenyl) diphosphate synthase, subunit 1 (PDSS1 or COQ1)	2e-72	C24A11.9	*coq-1*	Slow growth, uncoordinated, extended life span (RNAi); larval development arrest, slowed pharyngeal pumping, eventual paralysis and cell death (mutant)	[18,19,32]
11	4-hydroxybenzoate polyprenyltransferase (COQ2)	2e-83	F57B9.4	*coq-2*	Extended life span (RNAi); larval development arrest, slowed pharyngeal pumping, eventual paralysis and cell death (mutant)	[18,19]
12	Dehydrodolichyl diphosphate synthase (DHDDS)	7e-55	T01G1.4		No data	
13	Polyprenol reductase or steroid 5 alpha-reductase 3 (SRD5A3)	6e-14	B0024.13		No RNAi phenotype	[31,33]
14	Farnesyltransferase, CAAX box, subunit alpha (FNTA)	1e-55	R02D3.5	*fnta-1*	Protruding vulva, egg-laying abnormal (RNAi)	[31,32]
15	Farnesyltransferase, subunit beta (FNTB)	3e-90	F23B12.6	*fntb-1*	No phenotype (RNAi)	[31,33]
16	All-trans-GGPP synthase or geranylgeranyl diphosphate synthase	1e-6/5e-05	C24A11.9/R06C1.2	*coq-1 and fdps-1 are the closest homologs*	See *coq-1 *and *fdps-1*	
17	Protein geranylgeranyl transferase type-1 subunit beta (PGGT1B)	3e-73	Y48E1B.3		Larval lethal, larval arrest, postembryonic development abnormal (RNAi)	[31,33]
18	Rab geranylgeranyltransferase type 2 subunit alpha (RABGGTA)	5e-55	M57.2		Larval lethal, larval arrest, slow growth and induction of unfolding protein response (UPR)(RNAi)	[7,31,32]
19	Rab geranylgeranyltransferase type 2 subunit beta (RABGGTB)	1e-110	B0280.1	*ggtb-1*	Abnormal early embryo, developmental delay (RNAi)	[33]
20	Farnesyl-diphosphate farnesyltransferase 1 (FDFT1)	none	none		N/A	N/A

Unfortunately, besides whole-genome RNAi approaches, most enzymes of the main trunk have not been studied genetically: isopentyl diphosphate isomerase is the only main trunk enzyme for which there is a mutant, called *idi-1*. This mutant is likely a hypomorph since its phenotype is milder than the embryonic lethality observed in RNAi-treated animals, and consists of larval paralysis and lethality, and defect in engulfment of apoptotic corpses [8].

While the enzymes in the main trunk of the pathway are essential, the experimental evidence reviewed below so far suggests that none of the sub-branches are in themselves strictly essential for viability in the laboratory; it is likely that essential functions within these pathways are robust due to genetic redundancy. For example, isoprenylation of proteins may be achieved by three types of prenyl transferase complexes that may exhibit some promiscuity in their substrate specificities. We now turn our attention to each of the five biosynthetic pathways that branch off the main trunk of the mevalonate pathway.

### 2.2. Isopentyl adenosine

Isopentenyl phosphate, of which the synthesis is catalyzed by diphosphomevalonate decarboxylase, can be added to adenosine, producing isopentenyl adenosine. When present in tRNA, isopentenyl (i6A) derivatives are found at position 37 of tRNAs that read codons starting with U [9]. Modified nucleotides in tRNAs play an important role in stabilizing codon-anticodon interactions during translation, which is necessary to prevent shifts of the translational reading frame. For example, the hypermodified nucleoside 2-methylthio-N6 isopentenyl adenosine at position 37 (ms2i6A37) in tRNA^Phe^_GAA _stabilizes mRNA-tRNA interactions in all three tRNA binding sites [9,10]. In *C. elegans, gro-1 *is the homolog of the human gene tRNA isopentenyldiphosphate transferase (TRIT1). Mutations in this gene result in deregulated developmental, behavioral and reproductive rates, as well as increased life span [11]. One likely interpretation of these phenotypes is that reduced fidelity of translation causes inefficiency of several processes, resulting in a variable slow down of cellular events, which may contribute to the increased life span. This explanation is supported by the observation that mutations in other tRNA modifying enzymes also cause developmental delays and decreased reproductive capacity, especially at the high growth temperature of 25°C which should further weaken codon-anticodon interactions during translation [12]. In summary, tRNA modification appears to be an important but not strictly essential process of which the main function is to improve the fidelity of translation in cases where the codon-anticodon interaction involves a U-A pairing.

### 2.3. Coenzyme Q

**Figure 2:**
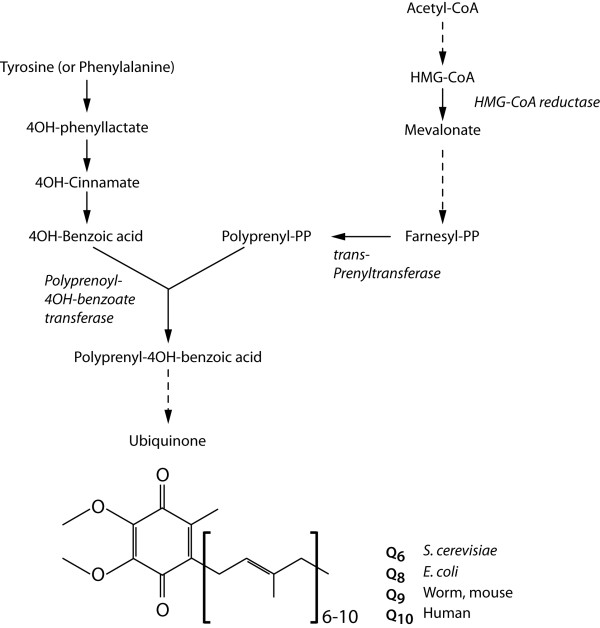
**Overview of the coenzyme Q biosynthetic pathway**. The key step in the pathway is the condensation reaction of the polyisoprenoid side-chain from the mevalonate pathway with 4-hydroxybenzoate, which is the product of a separate multi-step pathway starting from the precursors tyrosine or phenylalanine. Since these precursors are in excess compared to the polyisoprenoids, the rate of ubiquinol synthesis is determined by the availability of the polyisoprenoid: the conversion of Farnesyl-PP to polyprenyl-pp that is catalyzed by enzyme trans-prenyl transferase is the rate limiting step in the synthesis of ubiquinol. Ubiquinol consists of a modified benzoquinone ring attached to hydrophobic isoprenyl tail that contains 6-10 isoprenyl units depending on species. Doted lines represent additional enzymatic steps in the pathway.

The enzyme trans-prenyl transferase converts FPP to polyprenyl-PP and is the limiting step in the synthesis of Coenzyme Q (CoQ), also known as ubiquinol. CoQ consists of a modified benzoquinone ring that can be reversibly reduced and oxidized, and a hydrophobic isoprenyl tail that contains 6-10 isoprenyl units (Figure 2). The isoprenyl tail length of CoQ is species-specific, being 10 (CoQ10) in humans, nine (CoQ9) in rodents and *C. elegans*, and eight (CoQ8) in *E. coli*. CoQ is an active molecule that functions as an electron carrier in the mitochondrial electron transport chain. Electron transfer via CoQ involves the formation of semiubiquinone radicals, which cause the generation of superoxide radicals upon reaction with oxygen. In the reduced form, CoQH2 functions as a lipid-soluble antioxidant, and protects cells from lipid peroxidation. Thus, CoQ is also important as a lipophilic regulator of oxidative stress [1]. Inhibition of HMG-CoA reductase using statins in human causes a measurable decrease in serum levels of CoQ, which correlates with a decrease in cardiac function that can be reversed by providing CoQ as a dietary supplement [13,14].

CoQ is essential for development and survival of *C*. elegans since mutants unable to synthesize CoQ and grown on a bacterial food source lacking CoQ arrest during embryognesis or emerge from dauers as sterile adults [15,16]. A role for CoQ in regulating the timing of developmental events first became apparent when the gene *clk-1 *was shown to encode the worm homolog of COQ7, an enzyme important for the biosynthesis of CoQ [17]. *clk-1 *mutants grown on OP50, which provides some amount of nutritional CoQ, have deregulated developmental timing, resulting on average in a slower development and increased longevity. Similarly, RNAi knock-down of several enzymes involved in the synthesis of CoQ also cause extended life span in worms [18,19]. It has since become apparent that inhibition of mitochondrial respiration or ubiquinol production in *C. elegans *causes increased expression of cell-protective and metabolic genes as well as increased abundance of mitochondrial DNA, resulting in a slowing down of behavioral rates and extended lifespan [20]. These changes in gene expression have been called the ''retrograde response'' since they rely on a reversal in the normal direction of information flow between the mitochondria and nucleus [21]. The retrograde response may be a compensatory reaction to the normal decline in mitochondrial function seen with age since it is typically observed in older cells [21].

Besides its effects on biological rates and longevity, CoQ contributes to the robustness of specific developmental processes [22]. For example, the hypodermis is abnormal in *coq-8 *mutants, with evidence of extracellular matrix degeneration. The gonad also develops abnormally, and germ line viability and embryonic development exhibit failure rates with a penetrance varying from 2 to 40%, which suggests that CoQ provides an ancillary rather than critical function. Depressed levels of CoQ could result in limiting levels of ATP and pyrimidines with consequences on several processes [22].

### 2.4. Dolichols

The polyisoprenoid alcohols (dolichols and polyprenols) are found in all living organism, from bacteria to mammals [23]. In animal and yeast cells polyisoprenoids are derived from the cytoplasmic mevalonate pathway [24,25], and most polyisoprenoids in animals are dolichols: a group of long-chain mostly unsaturated organic compounds that are made up of varying numbers of isoprene units terminating in an α-saturated isoprenoid group, containing an alcohol functional group. The key enzymes of dolichol synthesis are cis-prenyltransferases (CTPs), responsible for the construction of the long hydrocarbon skeleton. CPTs elongate a short precursor, FPP, by sequential addition of the desired number of IPP molecules that results in formation of a stretch of cis units.

**Figure 3:**
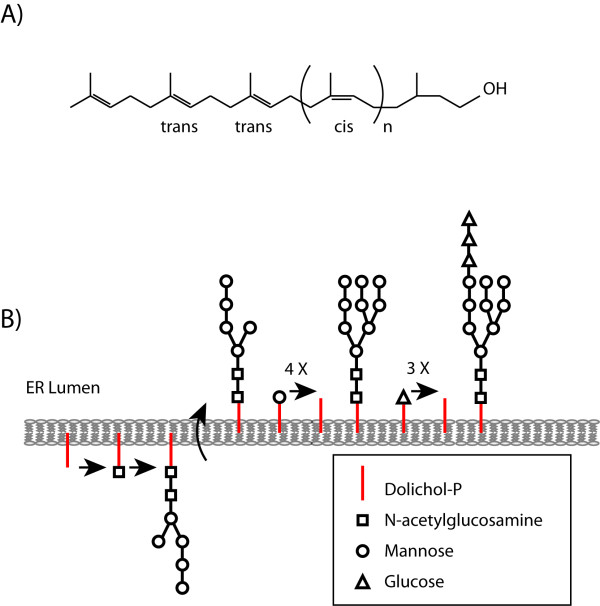
**Structure of Dolichol and their function during protein N-glycosylation**. **(A) **Structure of Dolichol, where *n *is dependent on particular cis-prenyltransferase. Dolichol phosphate is an isoprenoid compound (90-100 carbons total) made from dolichol by phosphorylation. Dolichol phosphate performs important functions in synthesis of N-linked glycoproteins, as illustrated in **(B)**. Dolichol phosphate is the structure upon which the complex oligosaccharide is made before transfer to the target protein. The addition of the first moiety, N-acetylglucosamine from UDP-N-acetylglucosamine, can be blocked by the antibiotic, tunicamycin. After assembly of the oligosaccharide is complete, the carbohydrate structure is transferred from dolichol phosphate to an asparagine residue of a target protein having the sequence Asn-x-Ser/Thr, where × is any amino acid. As also shown in (B), Dolichol phosphate can also act as a carrier of sugars to oligosaccharide chain synthesis assembly; such activated sugars include dolichol-P-mannose and dolichol-P-glucose.

Dolichols, which may be thought of as extremely hydrophobic superlipids, are postulated to be involved in the intracellular traffic of proteins and in cellular defense against adverse environmental conditions. Dolichols also play a role in protein N-glycosylation: the assembly of N-linked oligosaccharides in eukaryotes is initiated by the transfer of GlcNAc 1-P from UDP-GlcNAc to a dolichol-phosphate (Dol-P; specifically dolichyl-phosphate) forming GlcNAc-P-P-Dol (See Figure 3) [26]. Dol-P is itself produced by polyprenol reductase and, not surprisingly, there is a direct link between the dolichol biosynthetic pathway and congenital disorders of glycosylation [27-30]. During N-glycosylation, Dol-P functions as a membrane anchor for the formation of the oligosaccharide Glc3-Man9-GlcNAc2 (where Glc is glucose, Man is mannose, and GlcNAc is N-acetylglucosamine). This oligosaccharide is transferred from the dolichol donor onto certain asparagine residues of newly formed polypeptide chains. Dolichols are also involved in the transfer of monosaccharides to the forming Glc3-Man9-GlcNAc2-Dolichol carrier. In addition, dolichols can be adducted to proteins as a posttranslational modification where they act as cofactors for protein glycosylation in eukaryotes specifically for the biosynthesis of N- and O-glycoprotein and GPI-anchor [23].

The importance of dolichol biosynthesis in *C. elegans *is not very clear. There is no data available for mutations or RNAi knock-down of dehydrodolichyl diphosphate synthase, which converts FPP to polyprenyl-PP and is the first step of the dolichol biosynthesis branch that emerges from the main trunk of the mevalonate pathway. RNAi knock-down of the next enzyme, polyprenol reductase, causes no detectable phenotype (Table 1). Does that mean that protein glycosylation is unimportant in *C. elegans*? No. Some 1 465 *N*-glycosylated sites on 829 unique proteins have been identified in a proteomics approach [31]. Many of these N-glycosylated proteins are essential (e.g. the tyrosine protein kinase *let-23*) or would give visible phenotypes if inhibited (e.g. cell guidance genes such as *unc-5 *and *sax-7*), and it is simply not realistic to imagine that N-glycosylations do not contribute to important functions in any of these proteins. Furthermore, RNAi against enzymes involved in later N-glycosylation steps do cause severe phenotypes. For example RNAi knockdown of the worm homolog of the oligosaccharide transferase subunit STT3B (corresponding to the *C. elegans *ORF T12A.2) causes abnormal embryos, some of which grow into thin or long adults [32-34]. It is possible that the results of RNAi against the polyprenol reductase provide an incomplete picture regarding the importance of protein glycosylation in *C. elegans *because there are alternative pathways for dolichols synthesis [27].

### 2.5. Isoprenylated proteins

FPP can be converted to geranylgeranyl diphosphate (GGPP) by the enzyme trans-prenyl transferase. Many proteins owe their membrane association to their conjugation with FPP or GGPP moieties at their carboxyl terminus, a reaction catalyzed by dimeric farnesyl-protein transferases (FT) and geranylgeranyl-protein transferases (GGT1 and GGT2), respectively [35].

Isoprenylation of many polypeptides in *C. elegans *has been documented chemically [36]. Furthermore, isoprenylation is essential in *C. elegans*: treatment with gliotoxin, a prenylation inhibitor [37,38], causes lethality within 20 hours [36]. Querying PRENbase, a database for protein CaaX farnesylation, CaaX geranylgeranylation and Rab geranylgeranylation, for all known or likely *C. elegans *proteins that are prenylated either by FT, GGT1 or GGT2 produces a list of 49 *C. elegans *proteins falling into 9 clusters (See Additional file 1, Table S1). As expected, the three largest types of prenylated proteins in *C. elegans *are small GTPases: rab-like (28 proteins), ras-like (9 proteins) and rho/rac-like (6 proteins). The rab-like proteins are predicted to be mostly geranylgeranylprenylated by GGT2, while the ras-like and rho/rac-like may be prenylated by either GGT1 or FT. Many of the rab-like proteins are essential for embryogenesis and viability, four of the rho/rac-like proteins are essential (CED-10, CDC-42, RHO-1 and RAC-2) and only two of the ras-like proteins are essential (LET-60 and RHEB-1). The fact that many small GTPases implicated as oncogenes in human cancers are dependent on farnesylation or geranylgeranylation for their activity has led to the suggestion that statins could be effective anticancer agents since inhibition of HMG-CoA reductase would deplete the availability of the lipid moieties [35,39]. Importantly, *C. elegans *is also a relevant model in this context since statin treatment resulted in loss of prenylation of a CaaX-tagged GFP reporter, as well as causing growth arrest as previously mentioned (6).

None of the RNAis against the alpha or beta subunits of FT, GGT1 or GGT2 individually cause lethality (Table 1), while RNAi against specific prenylated small GTPases often does cause lethality (Additioanl file 1, Table S1). This means either that prenylation of small GTPases is not essential to their activities, or that the prenyltransferases can act redundantly on important substrates.

Besides the small GTPases, a small number of other proteins may be prenylated by FT or GGT1 in *C. elegans *(Additional file 1, Table S1), but this has not been demonstrated experimentally except for the nuclear lamin. Aging *C. elegans *exhibit characteristic changes in nuclear morphomlogy that are dependent on prenylated nuclear lamin, and prevented by the use of the prenylation inhibitors gliotoxin or manumycin [40].

### 2.6. Cholesterol

Cholesterol is an important component of many animal membranes, where it has significant effects on membrane properties, and is also a precursor to many steroid derivatives, including several hormones. *C. elegans *is a cholesterol auxotroph and it has no homologue for the enzyme squalene synthase (also known as farnesyl-diphosphate farnesyltransferase 1, or FDFT1). Therefore *C. elegans *does not use the mevalonate pathway to synthesize squalene or its derivative, cholesterol. Although cholesterol is not synthesised by *C. elegans*, it is essential for its survival, and is required for various biological processes, namely molting, reproduction, dauer formation and metabolism [41-44]. In the wild, *C. elegans *obtains cholesterol from its diet; in laboratory conditions, worms obtain it from bacteria and from the culture media, which is supplemented with cholesterol. In cholesterol-deprived conditions, the first generation from a cholesterol-fed mother grows and produces progeny normally, whereas the following generation arrests as L2 larvae [42,45]. These arrested larvae recover and follow normal development when provided with the final product of sterol the biosynthesis pathway, but not if intermediates are supplied, again demonstrating that *C. elegans *lack cholesterol synthesis machinery [46]. These findings also indicate that sterol is required in minor amounts for worm survival, and that it is provided via oocytes to the progeny. The main sterol transporting proteins in *C. elegans *are vitellogenins, homologues of mammalian apolipoproteins; these proteins are required for transporting sterols from the intestine to the oocytes via RME-2, a vitellogenin receptor [47,48]. The *C. elegans *sterol distribution has been studied using dehydroergosterol (DHE), a fluorescent analogue of cholesterol, which can functionally replace it. DHE accumulates in only small subsets of cells, namely nerve ring cells, pharynx cells, excretory gland cells, intestinal cells, oocytes and spermatozoa [45,49,50]. It is estimated that sterols in *C. elegans *lipid extracts are 20 times less abundant than in mammalian cells [51]. This reduced amount may be sufficient for the cell membrane of these specialized cells, and it is possible that the remaining cells do without cholesterol in their membranes. Note however that caveolin-1, a *bona fide *caveolae protein typically present in cholesterol-rich micro domains in plasma membrane, is found in *C. elegans *membrane extracts, indicating that lipid rafts are formed in this organism [52].

The evidence that sterols in *C. elegans *are required as precursors for hormones and other biologically active compounds came from the fact that cholesterol-deprived worms have molting defects and resemble dauer larvae, a diapause stage adapted to harsh conditions. The vertebrate gp330/megalin/LRP-2 protein homologue in worms, *lrp-1*, is required for molting and likely functions as a sterol receptor in hypodermal cells since megalin is an LDL receptor-related protein required for absorption of vitamin D [53].

Finally, work on two *C. elegans *genes, *daf-9 *and *daf-12*, suggests that sterols are also involved in dauer formation. *daf-9 *encodes a cytochrome P450 of the CYP2 subfamily that produces the dafachronic acid that promotes dauer entry by inhibiting *daf-12*, a nuclear hormone receptor [54-57]. Mutations in two other genes also indicate a role for sterols in regulating dauer formation: the *ncr-1 *and *ncr-2 *(*C. elegans *Niemann-Pick C1 protein homologues 1 and 2) are predicted to be involved in intracellular sterol trafficking, as with their human homologues which are required for intracellular cholesterol homeostasis [58]; *ncr-1;ncr-2 *double mutants constitutively form dauers.

## 3. Regulation of the mevalonate pathway

In eukaryotes, the mevalonate pathway is regulated at the transcriptional, translational and post-translational levels [59]. In mammals and yeast, sterols are modulate the function of various enzymes of the mevalonate pathway, depending on their concentration in the cell. The rate-limiting enzyme of the mevalonate pathway, HMG-CoA reductase (HMGR), is tightly regulated to control lipid homeostasis in the cell [60]. This regulation is achieved by sterol-mediated feedback inhibition to maintain appropriate levels of sterols and other branch-specific end-products in the cell [59]. In cholesterol-replete cells, the transcription factor SREBP (sterol regulatory element-binding protein) binds to the integral membrane protein Scap (sterol regulatory element-binding protein cleavage-activating protein) in the ER and becomes inactive. When cholesterol is depleted, this complex is transported to the Golgi apparatus, where SREBP undergoes proteolytic events resulting in its activation. The activated protein is transported to the nucleus and activates HMGR transcription [61]. Another important protein is Insig (insulin-induced gene), which in cholesterol-replete cells targets HMGR for proteolytic degradation, alone or with SREBP [62,63]. Insig-dependent HMGR degradation is also mediated by isoprenoids (geranylgeraniol; FPP) and these molecules also regulate HMGR at the translational level, although the molecular mechanisms behind this phenomenon are not clear [59,64-67]. Besides HMGR, sterols also regulate the expression of the HMG-CoA synthase and the LDL-receptors at the transcriptional level to maintain their optimal concentration in the cell.

The absence of the cholesterol synthetic branch in the *C. elegans *mevalonate pathway opens the possibility that the pathway in worms may be regulated differently than in mammalian and yeast cells: one would expect the products from the non-cholesterol branches to regulate the pathway, rather than the levels of sterols. However, this has not been addressed experimentally. It is however interesting to note that while *C. elegans *does contain homologues of SREBP and SCAP, it lacks an Insig gene [68]. The *C. elegans *SREBP homologue (*sbp-1*) is required for fat storage and regulates genes involved in fatty acid synthesis similarly to [69,70], but it is not known if it also regulates the mevalonate pathway.

## 4. Considerations regarding the Km for entry into the different branches of the pathway

To determine the severity with which each branch of the pathway would be affected if the common trunk were inhibited, as would occur upon statin treatment, it may be useful to know the Km of the enzymes involved. While this has not been determined in *C. elegans*, some information exists for mammalian cells. The Kms, some of which were reviewed elsewhere [71], are shown in Table 2. Given the Kms of the different steps, it seems that as mevalonate becomes limiting in mammalian cells, the branches to be affected would be first the synthesis of cholesterol (via squalene), then the geranlygeranylation of proteins, then the production of CoQ, and finally and only when mevalonate becomes almost completely depleted, the farnesylation of proteins. No information on the Km involved in the conversion of IPP to Dolichol-P could be found, and the Km of 0.4 μM for the CoQ branch is very much uncertain, as it is based on a rough cell assay. If these Kms are roughly preserved in *C. elegans*, one expects the bulk of the producs of the mevalonate pathway to be utilized for the production of geranylgeranyl-diphosphate and of CoQ.

**Table 2:** Estimated Km values for limiting steps in the mevalonate pathway.

Reaction	Km	Reference
FPP - > Squalene	2 μM	[80]
FPP- > GGPP	1 μM	[81]
FPP- > CoQ	0.4 μM	[82]
FPP- > Protein Farnesylation	5 nM	[83]

## 5. Experimental approaches to delineate key components and their biological roles

In mammals, the cholesterol synthesis sub-branch of the mevalonate pathway has been the most intensely studied due to the proposed involvement of cholesterol in atherosclerosis. Since this sub-branch is absent in *C. elegans*, this organism may be eminently suitable to study the non-cholesterol sub-branches of the pathway. Experimentally, this problem can be tackled in several ways, the most common being: 1) RNAi knock-down or mutation of the genes encoding the enzymes of the pathway; 2) Use of inhibitors to specifically block the whole pathway or individual sub-branches; and 3) Metabolic rescue of the individual sub-branches in the context of animals where the main trunk is inhibited.

In the first case, each enzyme of the pathway is knocked down in turn by feeding with bacteria expressing double stranded RNA (dsRNA) against them or by isolating deletion mutants. RNAi can be done broadly, for all the enzymes in the route, or specifically for the rate-limiting enzymes such as the HMG CoA reductase or *trans*-prenyl transferase. This method is especially important to study genes in the mevalonate pathway that are essential for survival or genes for which mutants are not yet available. Previous studies have shown that RNAi against enzymes in the main truck leads to lethal phenotype in *C. elegans *but not RNAi against enzymes of sub-branches (Table 1 and Figure 1). This suggests that there is no single non-cholesterol sub-branch that is essential for survival or that there is genetic redundancy within one or more essential sub-branch.

A second approach to probe the mevalonate pathway is to block its main trunk or various sub-branches using inhibitors of the specific enzymes. Such inhibitors include statins that inhibit HMG CoA reductase, bisphosphonates that inhibit farnesyl diphosphate synthase, manumycin and gliotoxin that inhibit farnesyltransferase, and several others (Table 3). Treatment with many of these inhibitors, specifically statins, bisphosphonates, manumycin and gliotoxin, have been tested in *C. elegans *and were found to cause growth arrest and lethality [6,37,40]. Farnesyltransferase inhibitors (FTIs) have been shown to cause developmental defects and inhibition of protein prenylation in *C. elegans*. FTIs treatment also suppresses age dependent nuclear morphology defects and defects caused by an activated form of the *ras *protein (*let-60*) in *C. elegans *[37,40]. Similarly, we have previously shown that statins can be used to inhibit the mevalonate pathway in *C. elegans*, which not only caused lethality, but also caused loss of protein prenylation and induced the Unfolded Protein Response (UPR); these effects were rescued by providing downstream metabolites of the mevalonate pathway indicating the specific activity of the statins in *C. elegans *[6].

**Table 3:** List of inhibitors against enzymes of the mevalonate pathway.

Enzymes	Inhibitors	Ref
HMG-CoA synthase	Hymeglusin	[84]
HMG-CoA reductase	Statins	[6]
Mevalonate kinase	Farnesyl thiodiphosphate	[85]
Mevalonate diphosphate decarboxylase	6-fluoro-Mevalonate 5-diphosphate	[86]
Farnesyl diphosphate synthase	Alendronate, Ibandronate, Pamidronate	[87,88]
Geranylgeranyl diphosphate synthase	Digeranyl bisphosphonate	[89]
Farnesyltransferase	Manumycin, Gliotoxin	[36,37,40]

Mutagenesis screens for mutants that are resistant to inhibitors that block main trunk of the pathway (statins and bisphosphonates), or inhibit the sub-branches (manumycin; gliotoxin) are key to identify additional genes that are dependent on, or regulators of, this pathway. Genetic loci causing resistance can easily be identified in *C. elegans *either by genetic mapping approach or by whole genome sequencing. Once identified it is relatively straightforward to perform functional and genetic interaction studies in *C. elegans *because of its short life cycle, well-studied morphology and simple genetic makeup.

Another experimental approach is metabolic rescue of the different branches of the pathway by selectively providing branch specific metabolites (such as Coenzyme Q9; GGPP; FPP; IPP) to *C. elegans *where the main mevalonate branch is inhibited either by inhibitors like statins or by RNAi. This approach is not only helpful in identifying the major non-cholesterol branch but also to determine their phenotypic contribution.

## Conclusions

*C. elegans *uses the mevalonate to synthesize metabolites other than cholesterol that are important for various biological processes, ranging from translation fidelity to protein localization, protein *N*-glycosylation and energy homeostasis. Inhibiting the synthesis of these metabolites by blocking the key enzymes through specific enzyme inhibitors results in development arrest and embryonic lethality, demonstrating the importance of these metabolites for survival. The ready ability to manipulate different branches of the pathway in *C. elegans *using RNAi or inhibitors makes it an excellent model to study their biological roles and regulation.

Previous studies have already established that statins can inhibit the mevalonate pathway in *C. elegans *as it does in other eukaryotic organisms and this inhibition results in mislocalization of intracellular signaling molecules such as small GTPases (Rho, Ras, Rac and Rab) [72,73]. These small GTPases are key biological switches that regulate signaling in the cell and that require prenylation for their proper localization. Mutations in these GTPase are one of the many causes of tumor progression and metastases [74], and identifying GTPases that are regulated by the mevalonate pathway in *C. elegans *can be a way to understand the role of the mevalonate pathway in regulating the mammalian homologs, and contribute to the effort to develop therapeutic approaches to modulate their activity, especially in cancerous cells.

Numerous studies have shown that statins have broader beneficial effects besides regulating cholesterol levels; these include anticancer effects, suppressive effects on the development of protein aggregates, and anti-inflammatory effects [75-78]. However, little is known about the underlying genetic basis of these non-cholesterol effects. Therefore, identifying genes that are involved in statin resistance in *C. elegans *may contribute to our understanding of the non-cholesterol statin effects in higher organisms. Such a research effort will also help in recognizing genetic factors that are responsible for poorly understood statin side effects, such as myopathy, neuropathy and insomnia [79]. To summarize: studies of the mevalonate pathway in *C. elegans *could be of great aid in addressing important medical issues.

## List of abbreviations used

CoQ: Coenzyme Q; FPP: Farnesyl diphosphate; GGPP: Geranylgeranyl diphosphate; HMGR: Hydroxymethylglutharyl-coenzyme A reductase; IPP: Isopentenyl diphosphate.

## Competing interests

The authors declare that they have no competing interests.

## Authors' contributions

Both authors contributed equally to surveying the literature in the field, writing of the manuscript and preparation of the figures, and both authors read and approved the final manuscript.

## Supplementary Material

Additional file 1 **Table S1. Prenylated proteins in *C. elegans *according to PRENbase (as of Oct 2011)**. The protein classes are separately shaded, with the class-type indicated for the first member of each class in the table. Click here for file
